# Ensuring Continuity of Care: Effective Strategies for the Post-hospitalization Transition of Psychiatric Patients in a Family Medicine Outpatient Clinic

**DOI:** 10.7759/cureus.52263

**Published:** 2024-01-14

**Authors:** Soji Ojo, Tricia O Okoye, Seyi A Olaniyi, Victor C Ofochukwu, Maureen O Obi, Amarachi Sarah Nwokolo, Chinwe Okeke-Moffatt, Oluwatosin B Iyun, Etinosa A Idemudia, Okiemute R Obodo, Violet C Mokwenye, Okelue E Okobi

**Affiliations:** 1 Psychiatry, University of Texas Health Science Center at Houston, Dallas, USA; 2 General Medicine, Ambrose Alli University, Ekpoma, NGA; 3 Medicine and Surgery, Obafemi Awolowo University, Ile-Ife, NGA; 4 Medicine, Ebonyi State University, Abakaliki, NGA; 5 Medicine and Surgery, Hospital Corporation of America (HCA) Houston Healthcare Pearland, Pearland, USA; 6 General Practice, Federal Teaching Hospital, Owerri, NGA; 7 General Medicine, Madonna University, Calgary, CAN; 8 Psychiatry and Behavioral Sciences, Washington University School of Health Sciences, San Pedro, BLZ; 9 School of Public Health and Family Medicine, University of Cape Town, Cape Town, ZAF; 10 Psychiatry and Behavioral Sciences, North Vista Hospital, Las Vegas, USA; 11 Medicine, Windsor University School of Medicine, Cayon, KNA; 12 General Practice, University of Uyo, Uyo, NGA; 13 General Practice, National Hospital Abuja, Abuja, NGA; 14 Family Medicine, Larkin Community Hospital Palm Springs Campus, Miami, USA; 15 Family Medicine, Medficient Health Systems, Laurel, USA; 16 Family Medicine, Lakeside Medical center, Belle Glade, USA

**Keywords:** physicians' role, family medicine practice, challenges and barriers, transition of care, post-hospitalization, psychiatry patients, continuity of care

## Abstract

In healthcare, continuity of care is a crucial element, especially for patients in the field of psychiatry who have recently been discharged from a hospital. The shift from inpatient to outpatient care poses challenges for patients and healthcare providers, including openness to treatment, competing priorities, financial insecurity, concerns and dilemmas faced by patients regarding their post-hospitalization life after improvements in symptoms, lack of social support, poor patient-doctor relationships, lack of insight, and stigma associated with mental illness. Therefore, it is vital to employ effective strategies to ensure patients receive the required care and support during this transition.

This review delves into the significance of continuity of care for psychiatric patients post-hospitalization, effective strategies for the transition, and the challenges and barriers to implementation from the perspective of a family medicine practice. To analyze physicians' role in managing psychiatric patients post-hospitalization, we developed a comprehensive search strategy. This involved extracting relevant data, updates, guidelines, and recommendations. Our search spanned various online repositories, such as PubMed and Google Scholar, specifically focusing on US-based guidelines aligned with our objectives. The search was conducted using medical subject headings (MeSH) and combinations of "OR," "AND," and "WITH." We crafted keywords to optimize our search strategy, including psychiatric illness, post-hospitalization, follow-up, follow-up care, primary care follow-up, and guidelines.

Exploring online repositories yielded 132 articles, and we identified some guidelines that addressed our objectives. We established inclusion and exclusion criteria for our review and reviewed 21 papers. Post-hospitalization follow-up is a critical facet of psychiatric care, aligning with guidelines from the American Psychiatric Association and other relevant sources. Emphasizing continuity of care ensures a smooth transition from inpatient to outpatient settings, sustaining therapeutic momentum and minimizing the risk of relapse. This comprehensive approach involves careful medication management, regular mental health assessments, education on condition-specific coping strategies, and coordinated care between healthcare providers. It includes conducting risk assessments, safety planning, building social support and community integration, prompt post-hospitalization follow-up, and tailored treatment plans. Together, these measures enhance overall wellness for recently discharged patients. This holistic strategy tackles pressing short-term needs while facilitating long-term stability, promoting resilience and successful community reintegration, reducing readmission likelihood, and ultimately supporting sustained recovery.

## Introduction and background

The transition from hospital admission to outpatient care is a crucial phase in psychiatric treatment for people with mental illnesses, emphasizing the necessity of continuity of care for effective management [1]. This continuity post-hospitalization is essential for minimizing relapse and re-admission risk, making it a vital determinant of both short-term and long-term outcomes following inpatient psychiatric treatment [2]. Studies by Smith et al. reveal that 30% to 50% of individuals admitted to psychiatric hospitals fail to attend post-discharge appointments within 30 days, resulting in adverse outcomes like heightened suicide risk, relapse, homelessness, and criminal justice involvement [3]. Despite mixed evidence, various studies emphasize the significance of timely follow-up visits post-inpatient care in psychiatric units to reduce the risks of re-admission and relapse [4-6]. Acknowledging the critical impact of care continuity, researchers across numerous studies, institutions, and clinical recommendations have explored targeted initiatives to lower psychiatric re-admission rates by improving transitional care coordination [3].

Needs assessment

Persons with mental illnesses often experience frequent hospital admissions. Available recent data indicates that 51 million adults in the United States have had one or more mental disorders in the past year, with 22% of them getting hospital admission for mental illnesses [7]. Approximately 8.6 million annual inpatient hospital admissions involve a mental illness, constituting 32.3% of total hospital admissions [5-7]. Despite high re-admission rates, factors influencing these rates vary, including the type of mental disorder, compliance with the hospitalization care plan, access to care, poverty, and biopsychosocial factors like age and support systems [7]. Compliance with medication post-discharge, access to ancillary treatments, duration from discharge to continuity physician follow-up, and access to a physician after discharge also contribute to relapse and re-admission rates. The transition from inpatient to outpatient care poses challenges for patients, families, and physicians, with nearly half of psychiatric patients not receiving or attending appropriate outpatient care visits [8]. Challenges in outpatient care provision include patient-level barriers and facilitators, such as openness to treatment, competing priorities, and financial insecurity [9]. This transition may evoke a sense of loss, uncertainty, and fear, leading to the resurfacing and persistence of mental health symptoms.

Barriers to the continuity of care

Ideally, the recommendation is that after hospital discharge, psychiatric patients should follow up with psychiatrists in outpatient settings [6,8]. However, there is a growing disparity between the number of post-discharged patients in psychiatry and the number of available psychiatrists to conduct guideline-recommended follow-ups [6-9]. This shortage necessitates task shifting to other sufficiently trained physicians or task sharing with mental health professionals in integrated care models to fulfill similar follow-up roles. Another barrier to care continuity is patient hesitation to attend outpatient appointments and lack of readiness to engage with providers, partially due to stigma and fear of judgment. Such aspects dissuade mentally ill individuals from seeking or accepting aftercare following discharge from the hospital. In the United States, family physicians play a pivotal role in post-hospitalization collaborative care, aiming to reduce re-hospitalization and improve patient outcomes. They conduct outpatient follow-ups, link inpatient and outpatient care plans, reconcile medications, reinforce adherence, match individual care requirements, and reduce early re-admission rates [10]. Patients with comorbid severe mental illnesses and medical conditions or concurrent disorders require timely follow-up to monitor their mental health, prevent potential re-admission, and facilitate recovery. These patients with complex requirements have elevated hospitalization rates and healthcare costs, emphasizing the need for preventative measures [11-13].

Objectives

Given these considerations, the present study aims to evaluate the role of continuity of care post-hospitalization in reducing re-admission for persons with mental illness, focusing on the perspective of family physicians. This study will explore challenges faced by patients with mental illness during the post-hospitalization transition, discussing evidence-based strategies and interventions within family medicine outpatient clinics to ensure successful continuity of care. Drawing on scientific sources, literature, and study findings, the research underscores the significance of continuity of care in psychiatric patients’ post-hospitalization journey.

Methodology

**Figure 1:**
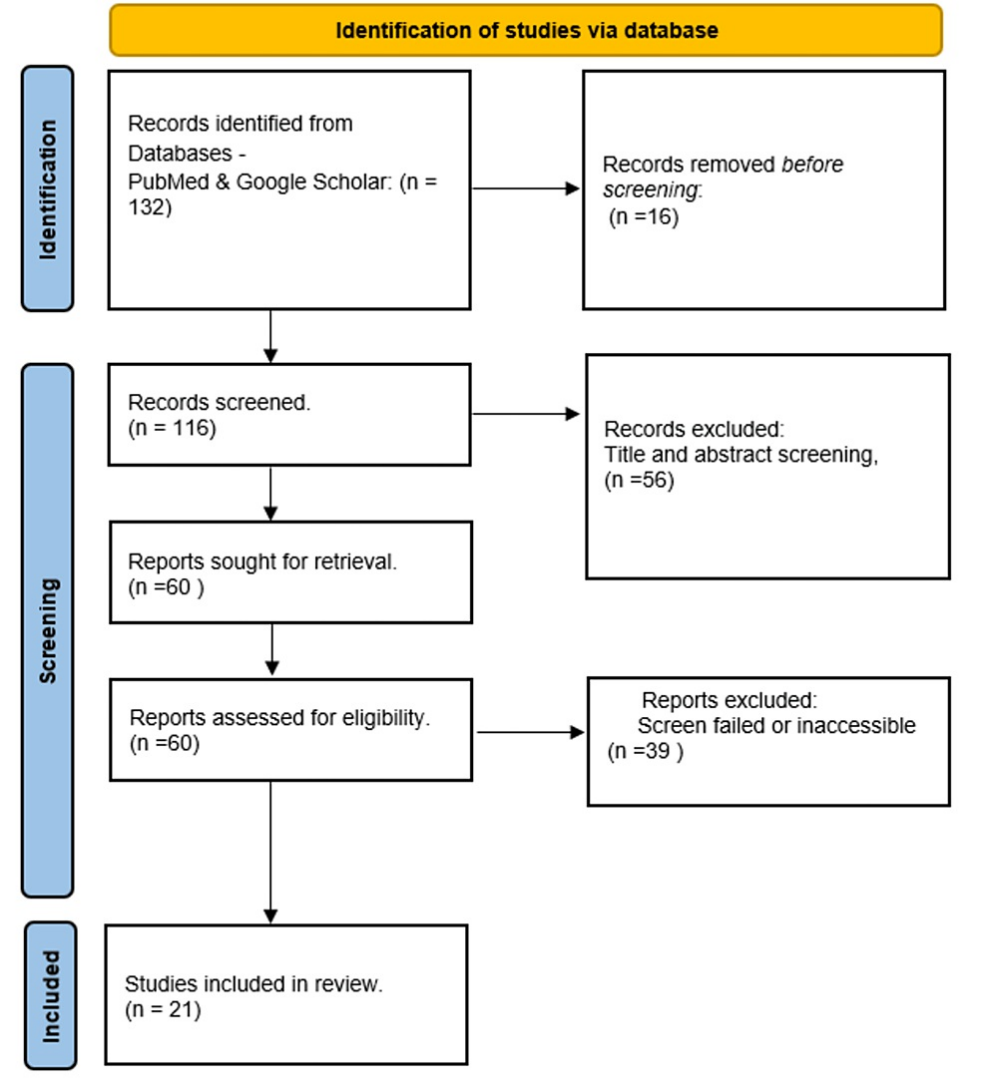
PRISMA workflow for included studies PRISMA: Preferred reporting items for systematic reviews and meta-analyses

This study’s methodology involved searching various medical databases, including PubMed and Google Scholar. The search strategy aimed to extract relevant data, updates, guidelines, and recommendations addressing the role of physicians in the effective management of post-hospitalized psychiatric patients. The researchers specifically searched for US-based guidelines related to the study objectives. Mesh terms “OR,” “AND,” and “WITH” were used, and effective keywords were generated to optimize the search strategy. The keywords included psychiatry illness, post-hospitalization, follow-up, follow-up care, and primary care follow-up guidelines. The search generated 132 articles, with only 21 meeting the inclusion criteria and addressing our objectives. To be included in this review, the literature had to satisfy the following criteria: journal-published studies and study protocols in English, with participants aged between 18 and 65 years, diagnosed with a mental illness, and discharged from a hospital psychiatric inpatient unit. Therefore, only original and review articles addressing the study’s objectives regarding the transition of care for psychiatric patients from hospitalization to primary care were included. The inclusion criteria also encompassed USA-based medical guidelines, WHO guidelines, and guidelines from prominent global psychiatry organizations. Studies focusing on organizational and structural levels of interventions and programs, as well as those on psychotherapeutic treatments with a specific focus on aspects like medication adherence and treatment for substance abuse, were also included (Figure 1).

The included studies were published between 2000 and 2023. Additionally, for studies to be included, the interventions used mainly focused on enhancing patient discharge from the hospital psychiatric inpatient unit to home, including pre-discharge and post-discharge aspects such as discharge plan development, pre-discharge and outpatient visit plans, needs assessment, and contact numbers for post-discharge care provision. Each component of the intervention had to originate in inpatient contexts. While randomization is desirable for minimizing study selection biases, it might not be practical in selecting studies for the objective of this paper. Consequently, the exclusion criteria encompassed studies published before 2000, opinion pieces, and newspaper articles. Furthermore, studies focusing on homeless persons and patients with mental or physical handicaps were excluded, including persons with difficulties talking or remembering things, those who have challenges sitting up, walking, and crawling, as well as those with challenges solving problems.

Quality and risk of bias assessment

To effectively assess the quality of the literature and studies included in this study, the CEPHOS-LINK quality assessment tool was employed. The assessment criteria incorporated in the tool included the target population’s representativeness in relation to the general psychiatric population, hospital readmission coverage, the completeness of the follow-up and participation rate, and the control for confounding aspects in statistical analysis. Every study selected for inclusion was individually evaluated by three reviewers, and any disagreement was resolved by an additional two researchers. Moreover, of the 21 studies included in this systematic review, 14 were found to have a low risk of bias, while three were found to have certain concerns. Still, three other studies had a high risk of bias. The risk of bias was mainly evident in non-randomized controlled trial (RCT) studies as well as studies with single-group designs, owing to the incomplete outcome data occasioned by higher drop-out rates and possible confounding factors that might not have been accounted for.

## Review

Initiating a comprehensive discharge planning process from the moment of hospital inpatient admission is essential for patients with mental illness to aim for goal-oriented, successful post-hospitalization follow-up that provides crucial care, support, and positive health outcomes [14]. To ensure the successful continuity of the care program post-hospitalization, it is imperative to consider and integrate numerous factors, including the type and severity of the mental disorder, patient compliance with the care plan, accessibility to post-discharge treatments, socioeconomic factors such as poverty, and the strength of the patient's support systems [14]. Lin et al.'s study further emphasizes key elements influencing patients' readmission to hospital psychiatric units. Their investigation delves into various discharge factors within hospitals and explores post-hospitalization aspects, including continuity of care, additional outpatient interventions, and patient follow-ups [15]. In alignment with these findings, it becomes evident that a comprehensive and strategic discharge process is pivotal not only for the immediate post-hospitalization phase but also for long-term outcomes in the community. This underscores the interconnectivity of discharge practices with patient outcomes beyond the hospital setting. Furthermore, examining the intricacies of Lin et al.'s study [15] offers valuable insights into tailoring interventions that address the multifaceted challenges faced by individuals with mental illness during and after the discharge process. Ensuring continuity of care for patients with psychiatric disorders during the transition from inpatient to outpatient settings is crucial for their well-being. Some effective strategies are summarized below in Table 1. By implementing these strategies, healthcare providers can enhance the continuity of care for individuals with psychiatric disorders, promoting a smoother transition from inpatient to outpatient settings [1-5,8,10-15].

**Table 1:** Ensuring continuity of care for patients with psychiatric disorders

Effective ways of facilitating the transition	How to facilitate the transition
Collaborative discharge planning	Involve both inpatient and outpatient mental health teams in the discharge planning process. Ensure open communication and coordination between the hospital staff and outpatient clinic professionals.
Comprehensive patient education	Educate the patient and their family about the importance of follow-up care and medication adherence. Provide information on available community resources and support services.
Timely scheduling of follow-up appointments	Schedule outpatient follow-up appointments before the patient is discharged. Ensure the first outpatient appointment is scheduled within a reasonable timeframe after discharge.
Medication management	Clearly communicate the patient's medication regimen to the outpatient provider. Provide an adequate supply of medications to bridge the gap until the first outpatient appointment.
Care coordination and case management	Assign a case manager or care coordinator to assist the patient during the transition. Facilitate communication between different healthcare providers and community support services.
Discharge summaries	Provide a detailed discharge summary that includes the patient's history, treatment plan, and recommendations for ongoing care. Share this summary with the outpatient provider to ensure continuity of care.
Family involvement	Engage family members in the discharge planning process, if appropriate, and with the patient's consent. Educate family members about signs of relapse and the importance of ongoing support.
Crisis intervention plan	Develop a crisis intervention plan outlining steps to take in case of a mental health crisis. Share this plan with the patient, family, and outpatient providers.
Transition support groups	Offer support groups or peer-led programs to help patients transition from inpatient to outpatient care. Provide a forum for patients to share their experiences and coping strategies.
Communication technology	Utilize secure electronic communication methods to share information between inpatient and outpatient teams. Enable virtual visits or telehealth options to facilitate ongoing contact and support.

Regulatory bodies, agencies, and scientific organizations have played a crucial role in shaping recommendations for effective post-hospitalization care. The Agency for Healthcare Research and Quality (AHRQ), the American Psychiatric Association, and the Association of American Family Physicians stand out with their proposed toolkit, offering a comprehensive approach to continuity of care and post-hospitalization follow-up. These recommendations not only emphasize clear statements but also advocate for a thorough analysis of readmission rates and the determination of specific goals. Implementation leadership, recognizing patients in need of follow-up, revising existing discharge workflows, allocating responsibilities, deciding on post-hospitalization care plans, and evaluating progress and execution are among the toolkit's essential components [16]. Moreover, a holistic perspective on post-hospitalization continuity of care is underscored by the suggestion that the program should encompass early screening and assessment, strategic discharge planning, well-coordinated discharge processes, effective implementation of discharge plans, and thoughtful post-hospitalization follow-up [16]. Building on this foundation, a separate study reinforces the idea that a comprehensive post-hospitalization follow-up plan is a multidisciplinary approach essential for the continuity of care [17]. This approach goes beyond a singular focus, recognizing the need for a collaborative effort involving various healthcare professionals and services. Donisi et al.'s proposal outlines an effective post-hospitalization care plan designed to enforce continuity of care and bridge the gap between hospital-based treatment and post-hospitalization care for patients with mental illness [17]. These recommendations and insights from regulatory bodies and studies emphasize the need for a nuanced and multidimensional approach to post-hospitalization care. By acknowledging the complexity of factors influencing patient outcomes, from initial screening to follow-up interventions, the healthcare system can enhance its ability to address the diverse needs of individuals with mental illness. Furthermore, these recommendations highlight the importance of coordination and collaboration among healthcare providers, ensuring a seamless transition and continuous support for patients as they move from hospital-based treatment to ongoing care in the community. The emphasis on multidisciplinary efforts echoes the recognition that mental health care requires a holistic and integrated approach, involving various specialties and services to provide comprehensive support for individuals managing mental health conditions.

In addition to these recommendations, scientific bodies emphasize the pivotal role of an effective discharge plan initiated during hospitalization [16,18]. This plan involves collaboration among the inpatient primary care team, social workers, patients, and their families to determine the most suitable post-hospitalization care. A range of options are considered, spanning rehabilitation centers, long-term care facilities, skilled nursing homes, subacute care homes, outpatient psychiatry care, or the involvement of a primary care physician, often family medicine physicians [16]. This collaborative effort ensures a patient-centric approach, tailoring the post-hospitalization care trajectory to individual needs and circumstances. Furthermore, effective strategies for the transition of psychiatric patients from inpatient to outpatient care are integral to successful continuity [15-18]. The collaborative care model emerges as a systematic strategy, integrating mental health specialists into primary care for a comprehensive approach to treating behavioral health conditions [18]. This model fosters a more holistic and accessible mental health care experience for patients within the primary care setting. Complementing this, transitional interventions, encompassing both pre-discharge and post-discharge phases, have demonstrated effectiveness in reducing readmission rates and enhancing overall patient outcomes [19]. These interventions bridge the gap between inpatient and outpatient care, addressing vulnerabilities during the critical transition period.

Transitional interventions in psychiatry serve as a crucial bridge between the intensive care provided in inpatient settings and the more flexible structures of outpatient care [1,18-24]. Notable examples of these transitional interventions include partial hospitalization programs (PHPs), where individuals engage in a structured, intensive treatment during the day yet have the freedom to return home in the evenings, fostering a connection with their community while receiving therapeutic support. Intensive outpatient programs (IOPs) offer a flexible step-down approach, allowing individuals to attend therapy sessions multiple times a week as they gradually reintegrate into their daily lives. Case management services play a pivotal role in coordinating resources, assisting with practical aspects such as housing and employment, and facilitating access to outpatient mental health services. Furthermore, the integration of medication management by home health care and follow-up ensures a smooth continuation of appropriate psychiatric care, including necessary adjustments and educational support regarding medication adherence. These personalized transitional interventions are designed to empower individuals in their recovery journey and facilitate successful reintegration into community living. Moreover, recognizing the significance of patient empowerment, patient-derived action plans play a crucial role in fostering self-efficacy and providing patients with a sense of control over their care [20]. These personalized plans take into account individual knowledge, resources, and preferences, aligning with the broader goal of patient-centered care [17-20]. By actively involving patients in their care decisions and providing them with tools to navigate their mental health journey, these action plans contribute to a more engaged and empowered patient population [20]. Overall, these strategies and models underscore the need for a comprehensive and tailored approach to post-hospitalization care, recognizing the diverse needs and preferences of individuals managing mental health conditions. The integration of these strategies into routine clinical practices holds the potential to significantly improve patient outcomes and reduce the risk of readmission.

Post-discharge activities are critical components of a comprehensive care strategy for patients with mental illness. Guidelines, such as those outlined by the National Committee for Quality Assurance Healthcare Effectiveness and Data Information Set (HEDIS), emphasize the importance of a timely post-hospitalization visit for psychiatric patients, ideally within 7 to 30 days after discharge [20,21]. This prompt follow-up is crucial for monitoring and addressing any emerging issues, contributing to the prevention of relapse and readmission. For those individuals transitioning to outpatient clinic care, a follow-up with a psychiatrist is typically expected. However, in instances where access to a psychiatrist is limited, family physicians can step in to provide essential care [20]. This collaborative approach aligns with the perspective advocated by Oldham et al., emphasizing the necessity for psychiatrists, family physicians, and other healthcare providers to collaborate closely. Their research highlights that a team-based outpatient care model, coupled with family engagement, contributes to a reduction in both hospital stay length and the overall cost of care [21]. In line with this collaborative approach, a study notes that an effective post-hospitalization care continuity program must be adaptable to meet the diverse needs of patients with mental illness [22]. The complexity of mental illnesses and the unique requirements of each patient necessitates a flexible and patient-centered approach. Moreover, this underscores the importance of inter-professional teamwork between psychiatrists and family physicians. Recognizing the intricate nature of mental health conditions, the study highlights the need for a cohesive and collaborative effort among healthcare professionals to provide holistic and tailored care [23,24]. This inter-professional teamwork not only addresses the multifaceted challenges faced by these individuals but also ensures that the care provided is comprehensive, addressing both the mental health condition and any co-occurring medical conditions. Overall, these insights underscore the significance of coordinated post-discharge activities that involve various healthcare professionals, promoting a patient-centered, adaptable, and inter-professional approach to enhance the effectiveness of post-hospitalization care for individuals with mental illness.

Several reports and studies in the United States have delved into the accessibility of psychological and pharmacological treatments in the post-hospitalization period [1]. Notably, research focusing on the correlation between post-hospitalization treatment and readmission rates revealed that individuals with mental illness receiving free or subsidized treatment were more prone to multiple readmissions compared to those without such benefits [25]. The duration of medication receipt also played a significant role, with non-hospitalized individuals reporting shorter treatment durations than their hospitalized counterparts, particularly in cases of schizophrenia [1]. Analyzing the effects of follow-ups within the first seven days post-hospitalization on readmission rates, it was found that having a family physician in the patient's community on the discharge day or a 24-hour follow-up was effective in minimizing readmission rates [1]. These findings underscore the importance of effective follow-up post-discharge and continuity of care conducted by family physicians in reducing hospital readmissions for patients with mental illness. However, a study by Pfeiffer et al. contradicted this, revealing that follow-ups within seven days post-hospitalization did not result in reduced readmission rates [25]. Other studies suggested that the existence of contact within the community at discharge, coupled with follow-ups by both psychiatrists and family physicians within seven days post-discharge, increased readmission rates [1,26]. Regarding follow-ups within 30 days post-hospitalization and their impact on readmission rates, increased contact was observed to considerably reduce readmission rates [27,28]. Interestingly, these correlations were found to be stronger in middle-aged and older patients compared to younger patients [1]. Conversely, a study on the voluntary readmission of patients with schizophrenia indicated that receiving follow-up services within 30 days following discharge increased the risk of readmission [29]. Several studies have also established a connection between continuity of care post-hospitalization for psychiatric patients and health outcomes. In a study on patients with mental illness enrolled in North Carolina Medicaid with schizophrenia and depression, a family physician follow-up within seven days post-hospitalization was associated with increased medication adherence compared to no follow-up within 30 days [30]. Similarly, a retrospective cohort study on bipolar and schizophrenia disorders revealed that follow-ups within 30 days post-hospitalization were linked to slightly lower hospital readmission risks [6]. Studies conducted in Japan and the United States echoed these findings, indicating that timely continuity of care post-hospitalization led to lower rates of hospital readmission [31]. Examining the correlations between early continuity of care in patients with mental illness post-hospitalization and the risk of suicide, recent population-based studies found that suicide risk was low among younger individuals aged between 10 and 18 years who received continuity of care within seven days post-discharge [32]. Conversely, a cohort study utilizing data from the Veterans Affairs Administrative Database revealed that psychiatric patients receiving at least two outpatient care visits within only one of the two months in the six months post-hospitalization presented an increased risk of suicide [30]. Despite these nuances, the heightened risks of readmissions and suicide among psychiatric patients lacking outpatient care post-hospitalization can be attributed to various challenges, as discussed below.

Challenges

Notable challenges in providing continuity of care for individuals with mental illness post-hospitalization include concerns and dilemmas faced by patients regarding their post-hospitalization life after improvements in symptoms. Factors such as lack of social support, poor patient-doctor relationships, lack of insight, and stigma often contribute to noncompliance with treatment programs and medications, subsequently worsening patients' symptoms and leading to harmful behaviors, suicide attempts, increased assault risk, extended hospital stays, impaired functioning, and a decrement in the quality of life [33]. The discharge juncture is recognized as a critical event and an integral aspect of the treatment process. Formal discharge planning results in the development of a personalized care program and support that addresses objectively evaluated patient needs at the point of discharge. Through discharge planning and continuity of care, necessary interventions, including cultural, educational, therapeutic, and social interventions, are implemented to safeguard and improve the well-being of patients with mental illness in outpatient contexts. Smith et al. emphasized that including outpatient appointments in discharge planning increased the odds of attending mental health services post-discharge, demonstrating the effectiveness of this routine discharge planning activity [3]. Other challenges and barriers to the implementation of continuity of care include fragmented care, a lack of provider cooperation, inadequate health awareness, and the stigma and discrimination faced by people with mental illness [34-36]. Challenges also exist in harnessing the benefits of integrated community mental health teams (CMHT) working to deliver continuity of care [35]. Addressing these challenges is crucial to ensuring that patients receive the necessary care and support.

Additional challenges in providing continuity of care post-hospitalization include a shortage of mental health providers, limited acceptance of Medicare by health providers, transportation barriers, ethnic and racial disparities in care provision, the low income of individuals with mental illness, and the inability of providers to contact homeless patients and those with unstable residences [1,13,31-35]. The stigma associated with mental illness acts as a significant barrier, leading to poor social support and hindering effective treatment-seeking behavior [8].

Moreover, the lack of direct or indirect communication between family physicians providing outpatient care and inpatient discharging care teams poses a significant challenge. Effective communication between these teams is crucial to ensuring the proper delivery of continuity of care. Although direct communication occurs in only about 20% of cases, the discharge summary is available in approximately 35% of initial follow-up appointments, underscoring the need for improved communication channels [37]. Timely follow-up and continuity of care, critical for preventing readmissions, have been identified as major challenges, with discharged patients often failing to see physicians within the recommended seven to 30 days post-discharge, resulting in deteriorating conditions and increased readmission rates [34-37].

The challenges identified highlight the need for evaluating and improving post-hospitalization transition programs to ensure patients receive the necessary continuity of care and support after discharge. Studies have assessed various interventions aimed at improving post-hospitalization treatment and support, including transitional and communication interventions at discharge [38]. Piette et al. explored a novel intervention for older adults with mental illness, while Becker et al. found that communication interventions at discharge have the potential to decrease hospital readmissions and improve treatment adherence [39,40]. Incentivizing higher-quality care has been shown to lead to better patient outcomes [41]. This ongoing evaluation and improvement are essential to ensuring patients receive the necessary care and support, preventing relapse and readmission. Notably, studies have revealed that timely post-discharge appointments with family physicians or psychiatrists significantly increase the likelihood of attending follow-up visits within seven to 30 days post-hospitalization. Integrating appointment scheduling into the discharge plan is likely to positively impact continuity of care in the initial days post-hospitalization, aligning with care standards that endorse appointments within seven days after discharge [37-39]. While routine discharge planning activities may not exert a lasting impact on treatment behaviors in the long run, considering various social, environmental, and clinical factors, the immediate benefits of well-timed post-discharge appointments are evident [39,41].

Furthermore, studies have shown the effectiveness of these strategies and disclosed significant correlations between continuity of care and inpatient use of care services, symptom scores, and reduced cost of care as a result of low readmission rates [10-15]. Various studies have also disclosed that improvement in continuity of care is linked to lower Medicaid costs [3,4]. Greater continuity of care has been linked to reduced costs in patients having more than one mental visit annually, in addition to being linked to a reduced probability of psychiatric hospitalization [8-13]. Outpatient expenditures remain a key predictor of the continuity of mental health care [7]. Additionally, continuity of care strategies, including the use of transition support groups, family involvement, and collaborative discharge planning, have been shown to reduce the cost of care [8-10]. Nevertheless, of the studies reviewed, none have indicated that continuity of care is associated with general satisfaction with health and life.

## Conclusions

The examination of post-hospitalization continuity of care for patients with mental illness from the lens of a family physician reveals a complex scenario characterized by challenges, interventions, and the crucial role of family physicians. Initiated from the point of hospital inpatient admission, continuity of care emerges as a participatory, continuous, and dynamic procedure vital for the well-being of individuals with mental illness. Noteworthy challenges, including patient-level barriers, a lack of social support, and stigma, impede the seamless transition from inpatient to outpatient care. Family physicians play a pivotal role in ensuring effective continuity of care amidst these challenges. Often leading outpatient follow-up, they connect inpatient and outpatient care plans, reconciling medications, ensuring adherence, aligning care with individual patient needs, and minimizing early readmissions. The collaborative care model, transitional interventions, and patient-derived action plans prove effective, emphasizing the significance of an integrated approach. Regulatory bodies and agencies, such as the AHRQ, offer valuable toolkits and recommendations for the implementation of effective post-hospitalization follow-up. These recommendations stress clear statements, readmission rate analysis, goal determination, leadership, patient recognition, discharge workflow revision, responsibility allocation, and care plan decisions. A comprehensive continuity of care program, suggested by various scientific bodies and regulators, encompasses early screening, discharge planning, coordination, implementation, and post-hospitalization follow-up.

Despite these interventions, challenges persist, from fragmented care to provider acceptance disparities and difficulties in scheduling timely follow-ups. Evaluating and improving post-hospitalization transition programs, incentivizing higher-quality care, and addressing these challenges are crucial for enhancing patient outcomes, preventing relapse, and minimizing readmission rates. This intricate web of challenges and interventions underscores the necessity for a comprehensive and integrated approach to post-hospitalization continuity of care for patients with mental illness. Family physicians, central to outpatient follow-up, must navigate these challenges to ensure a seamless transition from inpatient to outpatient care. The significance of effective communication, collaboration, and timely follow-up cannot be overstated.

Future research and practice should focus on addressing these challenges, refining interventions, and fostering a healthcare ecosystem that prioritizes the holistic well-being of individuals with mental illness. Moreover, it is important that a dedicated integrated practice unit be constituted to ensure effective and timely handling of the various challenges that the patient might be experiencing in accessing continuity of care post-hospitalization. Also, based on the findings of this review, it is recommended that inpatient psychiatrists, physicians, and family physicians jointly work towards ensuring adequate customization of integrated care interventions as a means of ensuring improvement of the quality of continuity of care. Further, it is recommended that physicians focus more on personalization of the treatment protocols utilized during continuity of care to not only optimize care outcomes but also reduce the cost of care. Ensuring effective communication is vital to improving the outcomes of continuity of care, as it ensures that the physician and the patients are able to communicate in real time and get challenges that might be preventing effective delivery of continuity of care resolved.

Lastly, several implications can be drawn from the findings of this review. For instance, given that continuity of care is a construct that is both indispensable and multidimensional, within a high-quality care context, continuity of care has become increasingly important with regard to the provision of psychiatric care owing to the recurrent and long-lasting episodes and various important treatment aspects. Thus, the findings of this review aim to inform the management of care continuity post-hospitalization by ensuring that care services are offered in a timely and complementary manner to offer a sense of security and predictability for psychiatric care in the future. Further, the findings will inform the need for relational continuity as they emphasize the need for the maintenance of continuing personal relationships between the psychiatric patient and the physician, or a consistent team of healthcare professionals. The findings of this review might also help inform policymakers, patients, and healthcare professionals in their efforts aimed at improving organizations, incorporating services, and orienting them towards achieving full recovery.
